# Shrinkage Clustering: a fast and size-constrained clustering algorithm for biomedical applications

**DOI:** 10.1186/s12859-018-2022-8

**Published:** 2018-01-23

**Authors:** Chenyue W. Hu, Hanyang Li, Amina A. Qutub

**Affiliations:** Department of Bioengineering, Rice University, Main Street, Houston, 77030 USA

**Keywords:** Clustering, Matrix factorization, Cancer subtyping, Gene expression

## Abstract

**Background:**

Many common clustering algorithms require a two-step process that limits their efficiency. The algorithms need to be performed repetitively and need to be implemented together with a model selection criterion. These two steps are needed in order to determine both the number of clusters present in the data and the corresponding cluster memberships. As biomedical datasets increase in size and prevalence, there is a growing need for new methods that are more convenient to implement and are more computationally efficient. In addition, it is often essential to obtain clusters of sufficient sample size to make the clustering result meaningful and interpretable for subsequent analysis.

**Results:**

We introduce *Shrinkage Clustering*, a novel clustering algorithm based on matrix factorization that simultaneously finds the optimal number of clusters while partitioning the data. We report its performances across multiple simulated and actual datasets, and demonstrate its strength in accuracy and speed applied to subtyping cancer and brain tissues. In addition, the algorithm offers a straightforward solution to clustering with cluster size constraints.

**Conclusions:**

Given its ease of implementation, computing efficiency and extensible structure, *Shrinkage Clustering* can be applied broadly to solve biomedical clustering tasks especially when dealing with large datasets.

## Background

Cluster analysis is one of the most frequently used unsupervised machine learning methods in biomedicine. The task of clustering is to automatically uncover the natural groupings of a set of objects based on some known similarity relationships. Often employed as a first step in a series of biomedical data analyses, cluster analysis helps to identify distinct patterns in data and suggest classification of objects (e.g. genes, cells, tissue samples, patients) that are functionally similar or related. Typical applications of clustering include subtyping cancer based on gene expression levels [1–3], classifying protein subfamilies based on sequence similarities [4–6], distinguishing cell phenotypes based on morphological imaging metrics [7, 8], and identifying disease phenotypes based on physiological and clinical information [9, 10].

Many algorithms have been developed over the years for cluster analysis [11, 12], including hierarchical approaches [13] (e.g., *ward-linkage*, *single-linkage*) and partitional approaches that are centroid-based (e.g., *K-means* [14, 15]), density-based (e.g., DBSCAN [16]), distribution-based (e.g., Gaussian mixture models [17]), or graph-based (e.g., Normalized Cut [18]). Notably, nonnegative matrix factorization (NMF) has received a lot of attention in application to cluster analysis, because of its ability to solve challenging pattern recognition problems and the flexibility of its framework [19]. NMF-based methods have been shown to be equivalent to a relaxed *K-means* clustering and Normalized Cut spectral clustering with particular cost functions [20], and NMF-based algorithms have been successfully applied to clustering biomedical data [21].

With few exceptions, most clustering algorithms group objects into a pre-determined number of clusters, and do not inherently look for the number of clusters in the data. Therefore, cluster evaluation measures are often employed and are coupled with clustering algorithms to select the optimal clustering solution from a series of solutions with varied cluster numbers. Commonly used model selection methods for clustering, which vary in cluster quality assessment criteria and sampling procedures, include *Silhouette* [22], *X-means* [23], *Gap Statistic* [24], *Consensus Clustering* [25], *Stability Selection* [26], and *Progeny Clustering* [27]. The drawbacks of coupling cluster evaluation with clustering algorithms include (i) computation burden, since the clustering needs to be performed with various cluster numbers and sometimes multiple times to assess the solution’s stability; and (ii) implementation burden, since the integration can be laborious if algorithms are programmed in different languages or are available on different platforms.

Here, we propose a novel clustering algorithm *Shrinkage Clustering* based on symmetric nonnegative matrix factorization notions [28]. Specifically, we utilize unique properties of a hard clustering assignment matrix to simplify the matrix factorization problem and to design a fast algorithm that accomplishes the two tasks of determining the optimal cluster number and performing clustering in one. The *Shrinkage Clustering* algorithm is mathematically straightforward, computationally efficient, and structurally flexible. In addition, the flexible framework of the algorithm allows us to extend it to clustering applications with minimum cluster size constraints.

## Methods

### Problem formulation

Let *X*={*X*_1_, …, *X*_*N*_} be a finite set of *N* objects. The task of cluster analysis is to group objects that are similar to each other and separate those that are dissimilar to each other. The completion of a clustering task can be broken down to two steps: (i) deriving similarity relationships among all objects (e.g., Euclidean distance); (ii) clustering objects based on these relationships. The first step is sometimes omitted when the similarity relationships are directly provided as raw data, for example in the case of clustering genes based on their sequence similarities. Here, we assume that the similarity relationships were already derived and are available in the form of a similarity matrix *S*_*N*×*N*_, where *S*_*ij*_∈[0,1] and *S*_*ij*_=*S*_*ji*_. In the similarity matrix, a larger *S*_*ij*_ represents more resemblance in pattern or closer proximity in space between *X*_*i*_ and *X*_*j*_, and vice versa.

Suppose *A*_*N*×*K*_ is a clustering solution for objects with similarity relationships *S*_*N*×*N*_. Since we are only considering the case of hard clustering, we have *A*_*ik*_∈{0,1} and $\sum _{k=1}^{K} A_{ik} =1$. Specifically, *K* is the number of clusters obtained, and *A*_*ik*_ takes the value of 1 if *X*_*i*_ belongs to cluster *k* and takes the value of 0 if it does not. The product of *A* and its transpose *A*^*T*^ represents a solution-based similarity relationship $\hat {S}$ (i.e. $\hat {S} = AA^{T}$), in which $\hat {S}_{ij}$ takes the value of 1 when *X*_*i*_ and *X*_*j*_ are in the same cluster and 0 otherwise. Unlike *S*_*ij*_ which can take continuous values between 0 and 1, $\hat {S}_{ij}$ is a binary representation of the similarity relationships indicated by the clustering solution. If a clustering solution is optimal, the solution-based similarity matrix $\hat {S}$ should be similar to the original similarity matrix *S* if not equal.

Based on this intuition, we formulate the clustering task mathematically as

1 $$  \begin{aligned}  & \underset{A}{\text{min}} \quad\quad\quad\quad ||{S-AA}^{T}||_{F}\\
 & \text{subject~to}  \quad\quad A_{ik} \in \{0,1\}, \quad {\sum\limits}_{k=1}^{K} {A}_{ik}=1, \quad {\sum\limits}_{i=1}^{N} {A}_{ik} \neq 0 \ . \end{aligned}  $$

The goal of clustering is therefore to find an optimal cluster assignment matrix *A*, which represents similarity relationships that best approximate the similarity matrix *S* derived from the data. The task of clustering is transformed into a matrix factorization problem, which can be readily solved by existing algorithms. However, most matrix factorization algorithms are generic (not tailored to solving special cases like Function 1), and are therefore computationally expensive.

### Properties and rationale

In this section, we explore some special properties of the objective Function 1 that lay the ground for *Shrinkage Clustering*. Unlike traditional matrix factorization problems, the solution *A* we are trying to obtain has special properties, i.e. *A*_*ik*_∈{0,1} and $\sum _{k=1}^{K} A_{ik}=1$. This binary property of *A* greatly simplifies the objective Function 1 as below.

$$ \begin{aligned} & \underset{A}{\text{min}} \|S-AA^{T}\|_{F}\\ &\quad = \underset{A}{\text{min}} \sum\limits_{i=1}^{N}\sum\limits_{j=1}^{N} (S_{ij}-A_{i} \bullet A_{j})^{2} \\ &\quad = \underset{A}{\text{min}} \sum\limits_{i=1}^{N} \left(\sum\limits_{j \in \{ j|A_{i}=A_{j} \}} (S_{ij} -1)^{2} + \sum\limits_{j \in \{ j|A_{i}\neq A_{j}\}} S_{ij}^{2}\right) \\ &\quad = \underset{A}{\text{min}} \left(\sum\limits_{i=1}^{N} \sum\limits_{j \in \{ j|A_{i}=A_{j} \}} (1-2S_{ij}) + \sum\limits_{i=1}^{N} \sum\limits_{j=1}^{N} S_{ij}^{2} \right) \end{aligned} $$

Here, *A*_*i*_ represents the *i*th row of *A*, and the symbol ∙ denotes the inner product of two vectors. Note that *A*_*i*_∙*A*_*j*_ take binary values of either 0 or 1, because *A*_*ik*_∈{0,1} and $\sum _{k=1}^{K} A_{ik} =1$. In addition, $\sum _{i=1}^{N} \sum _{j=1}^{N} S_{ij}^{2}$ is a constant that does not depend on the clustering solution *A*. Based on this simplification, we can reformulate the clustering problem as

2 $$ \begin{aligned}  &\underset{A}{\text{min}} f(A) = \sum\limits_{i=1}^{N}\sum\limits_{j \in \{ j|A_{i}=A_{j} \}} \left(1-2S_{ij}\right).\\ \end{aligned}  $$

Let’s now consider how the value of the objective Function 2 changes when we change the cluster membership of an object *X*_*i*_. Suppose we start with a clustering solution *A*, in which *X*_*i*_ belongs to cluster *k* (*A*_*ik*_=1). When we change the cluster membership of *X*_*i*_ from *k* to *k*^′^ with the rest remaining the same, we would obtain a new clustering solution *A*^′^, in which $A^{\prime }_{ik'}=1$ and $A^{\prime }_{ik}=0$. Since *S* is symmetric (i.e. *S*_*ij*_=*S*_*ji*_), the value change of the objective Function 2 is

3 $$ \begin{aligned}  \bigtriangleup f_{i} &:= f(A')-f(A) \\ & =\sum\limits_{j \in k'} \left(1-2S_{ij}\right)-\sum\limits_{j \in k}\left(1-2S_{ij}\right) + \sum\limits_{j \in k'}\left(1-2S_{ji}\right)\\ &\quad - \sum\limits_{j \in k}\left(1-2S_{ji}\right) \\ &=2\left(\sum\limits_{j \in k'} \left(1-2S_{ij}\right)-\sum\limits_{j \in k}\left(1-2S_{ij}\right)\right) \ \ \ . \end{aligned}  $$

### Shrinkage clustering: Base algorithm

Based on the simplified objective Function 2 and its properties with cluster changes (Function 3), we designed a greedy algorithm *Shrinkage Clustering* to rapidly look for a clustering solution *A* that factorizes a given similarity matrix *S*. As described in Algorithm 1, *Shrinkage Clustering* begins by randomly assigning objects to a sufficiently large number of initial clusters. During each iteration, the algorithm first removes any empty clusters generated from the previous iteration, a step that gradually shrinks the number of clusters; then it permutes the cluster membership of the object that most minimizes the objective function. The algorithm stops when the solution converges (i.e. no cluster membership permutation can further minimize the objective function), or when a pre-specified maximum number of iterations is reached. *Shrinkage Clustering* is guaranteed to converge to a local optimum (see Theorem 1 below).





The main and advantageous feature of *Shrinkage Clustering* is that it shrinks the number of clusters while finding the clustering solution. During the process of permuting cluster memberships to minimize the objective function, clusters automatically collapse and become empty until the optimization process is stabilized and the optimal cluster memberships are found. The number of clusters remaining in the end is the optimal number of clusters, since it stabilizes the final solution. Therefore, *Shrinkage Clustering* achieves both tasks of (i) finding the optimal number of clusters and (ii) finding the clustering memberships.

#### **Theorem 1**

*Shrinkage Clustering* monotonically converges to a (local) optimum.

#### *Proof*

We first demonstrate the monotonically decreasing property of the objective Function 2 in each iteration of the algorithm. There are two steps taken in each iteration: (i) removal of empty clusters; and (ii) permutation of cluster memberships. Step (i) does not change the value of the objective function, because the objective function only depends on non-empty clusters. On the other hand, step (ii) always lowers the objective function, since a cluster membership permutation is chosen based on its ability to achieve the greatest minimization of the objective function. Combing step (i) and (ii), it is obvious that the value of the objective function monotonically decreases with each iteration. Since ∥*S*−*AA*^*T*^∥_*F*_≥0 and $\big \|S-AA^{T}\big \|_{F}=\sum _{i=1}^{N} \sum _{j \in \{ j|A_{i}=A_{j} \}} \left (1-2S_{ij}\right) + \sum _{i=1}^{N} \sum _{j=1}^{N} S_{ij}^{2}$, the objective function has a lower bound of $-\sum _{i=1}^{N} \sum _{j=1}^{N} S_{ij}^{2}$. Therefore, a convergence to a (local) optimum is guaranteed, because the algorithm is monotonically decreasing with a lower bound. □

### Shrinkage clustering with cluster size constraints

It is well-known that *K-means* can generate empty clusters when clustering high-dimensional data with over 20 clusters, and *Hierarchical Clustering* often generate tiny clusters with few samples. In practice, clusters of too small a size can sometimes be full of outliers, and they are often not preferred in cluster interpretation since most statistical tests do not apply to small sample sizes. Though extensions to *K-means* were proposed to solve this issue [29], the attempt to control cluster sizes has not been easy. In contrast, the flexibility and the structure of *Shrinkage Clustering* offers a straightforward and rapid solution to enforcing constraints on cluster sizes. To generate a clustering solution with each cluster containing at least *ω* objects, we can simply modify Step 1 of the iteration loop in Algorithm 1. Instead of removing empty clusters in the beginning of each iteration, we now remove clusters of sizes smaller than a pre-specified size *ω*. The base algorithm (Algorithm 1) can be viewed as a special case of *w*=0 in the size-constrained *Shrinkage Clustering* algorithm.

## Results

### Experiments on similarity data

#### Testing with simulated similarity matrices

We first use simulated similarity matrices to test the performance of *Shrinkage Clustering* and to examine its sensitivity to the initial parameters and noise. As a proof of concept, we generate a similarity matrix *S* directly from a known cluster assignment matrix *A* by *S*=*AA*^*T*^. Here, the cluster assignment matrix *A*_100×5_ is randomly generated to consist of 100 objects grouped into 5 clusters with unequal cluster sizes (i.e. 15, 17, 20, 24 and 24 respectively). The similarity matrix *S*_100×100_ generated from the product of *A* and *A*^*T*^ therefore represents an ideal case, where there is no noise, since each entry of *S* only takes a binary value of either 0 or 1.

**Fig. 1:**
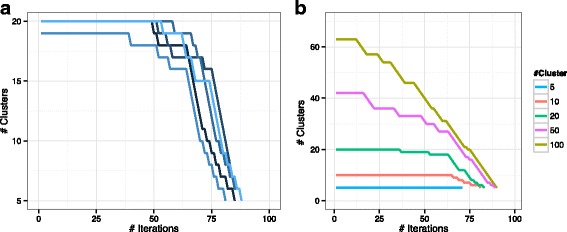
Performances of the base algorithm on simulated similarity data. Shrinkage paths plot changes in cluster numbers through the entire iteration process. **a** The first five shrinkage paths from the 1000 runs (with 20 initial random clusters) are illustrated. **b** Example shrinkage paths are shown from initiating the algorithm with 5, 10, 20, 50 and 100 random clusters

We apply *Shrinkage Clustering* to this simulated similarity matrix *S* with 20 initial random clusters and repeat the algorithm for 1000 times. Each run, the algorithm accurately generates 5 clusters with cluster assignments $\tilde {A}$ in perfect match with the true cluster assignments *A* (an example shown in Table 1 under *ω*=0), demonstrating the algorithm’s ability to perfectly recover the cluster assignments in a non-noisy scenario. The shrinkage paths of the first 5 runs (Fig. 1a) illustrate that most runs start around a number of 20 clusters, and all of them shrink down gradually to a final number of 5 clusters when the solution reaches an optimum.

**Table 1:** Clustering results of simulated similarity matrices with varying size constraints (*ω*), where C is the cluster generated by *Shrinkage Clustering*

True Label	*ω*=0				*ω*=20					*ω*=25	
	C1	C2	C3	C4	C5	C1	C2	C3	C4	C1	C2
Cluster 1	0	0	24	0	0	0	24	0	0	0	24
Cluster 2	15	0	0	0	0	15	0	0	0	15	0
Cluster 3	0	0	0	24	0	0	0	24	0	0	24
Cluster 4	0	17	0	0	0	17	0	0	0	17	0
Cluster 5	0	0	0	0	20	0	0	0	20	20	0

To examine whether *Shrinkage Clustering* is able to accurately identify imbalanced cluster structures, we generate an alternative version of *A*_100×5_ with great differences in cluster sizes (i.e. 2, 3, 10, 35 and 50). We run the algorithm with the same parameters as before (20 initial random clusters repeated for 1000 times). The algorithm generates 5 clusters with the correct cluster assignment in every run, showing its ability to accurately find the true cluster number and true cluster assignments in data with imbalanced cluster sizes.

We then test whether the algorithm is sensitive to the initial number of clusters (*K*_0_) by running it with *K*_0_ ranging from 5 (true number of clusters) to 100 (maximum number of clusters). In each case, the true cluster structure is recovered perfectly, demonstrating the robustness of the algorithm to different initial cluster numbers. The shrinkage paths in Fig. 1b clearly show that in spite of starting with various initial numbers of clusters, all paths converge to the same number of clusters at the end.

**Fig. 2:**
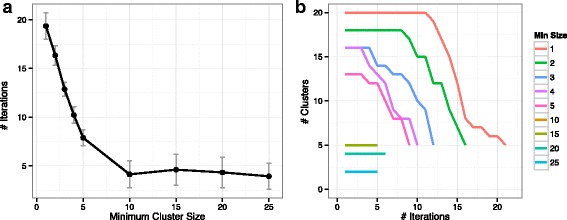
Performances of *Shrinkage Clustering* with cluster size constraints. **a** The average number of iterations spent is plotted with *ω* taking values of 1 to 5, 10, 15, 20 and 25. **b** Example shrinkage paths are shown for *ω* of 1 to 5, 10, 15, 20 and 25 (path of *ω*=10 is in overlap with *ω*=15)

Next, we investigate the effects of size constraints on *Shrinkage Clustering*’s performance by varying *ω* from 1 to 5, 10, 20 and 25. The algorithm is repeated 50 times in each case. We find that as long as *ω* is smaller than the true minimum cluster size (i.e. 15), the size constrained algorithm can perfectly recover the true cluster assignments *A* in the same way as the base algorithm. Once *ω* exceeds the true minimum cluster size, clusters are forced to merge and therefore result in a smaller number of clusters (example clustering solutions of *ω*=20 and *ω*=25 shown in Table 1). In these cases, it is impossible to find the true cluster structure because the algorithm starts off with fewer clusters than the true number of clusters and it works uni-directionally (i.e. only shrinks). Besides enabling supervision on the cluster sizes, size-constrained *Shrinkage Clustering* is also computationally advantageous. Figure 2a shows that a larger *ω* results in fewer iterations needed for the algorithm to converge, and the effect reaches a plateau once *ω* reaches certain sizes (e.g. *ω*=10 in this case). The shrinkage paths (Fig. 2b) show that it is the reduced number of iterations at the beginning of a run that speeds up the entire process of solution finding when *ω* is large.

**Fig. 3:**
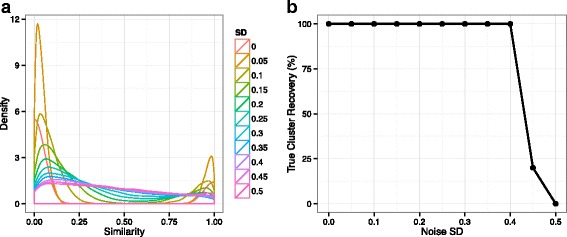
Robustness of *Shrinkage Clustering* against noise. **a** The distribution density of *S*^*N*^ is shown with a varying degree of noise, as *ε* is sampled with *σ* from 0 to 0.5. **b** The probability of successfully recovering the underlying cluster structure is plotted against different noise levels. The true cluster recovery is defined as the frequency of generating the exact same cluster assignment as the true cluster assignement when clustering the data with noise generated 1000 times

In reality, it is rare to find a perfectly binary similarity matrix similar to what we generated from a known cluster assignment matrix. There is always a certain degree of noise clouding our observations. To investigate how much noise the algorithm can tolerate in the data, we add a layer of Gaussian noise over the simulated similarity matrix. Since *S*_*ij*_∈{0,1}, we create a new similarity matrix *S*^*N*^ containing noise defined by

$$S_{ij}^{N} = \left\{ \begin{array}{ll} |\varepsilon_{ij}| & \quad \text{if}\ S_{ij}=0 \\ 1-|\varepsilon_{ij}| & \quad \text{if}\ S_{ij}=1 \end{array}, \right. $$

where *ε*_*ij*_∼*N*(0,*σ*^2^). The standard deviation *σ* is varied from 0 to 0.5, and *S*^*N*^ is generated 1000 times by randomly sampling *ε*_*ij*_ with each *σ* value. Figure 3a illustrates the changes of the similarity distribution density as *σ* increases. When *σ*=0 (i.e. no noise), *S*^*N*^ is Bernoulli distributed. As *σ* becomes larger and larger, the bimodal shape is flattened by noise. When *σ*=0.5, approximately 32% of the similarity relationships are reversed, and hence observations have been perturbed too much to infer the underlying cluster structure. The performances of *Shrinkage Clustering* in these noisy conditions are shown in Fig. 3b. The algorithm proves to be quite robust against noise, as the true cluster structure is 100% recovered in all conditions except for when *σ*>0.4.

#### Case Study: TCGA Dataset

To illustrate the performance of *Shrinkage Clustering* on real biological similarity data, we apply the algorithm to subtyping tumors from the Cancer Genome Atlas (TCGA) dataset [30]. Derived from the TCGA database, the dataset includes 293 samples from 3 types of cancers, which are Breast Invasive Carcinoma (BRCA, 207 samples), Glioblastoma Multiforme (GBM, 67 samples) and Lung Squamous Cell Carcinoma (LUSC, 19 samples). The data is presented in the form of a similarity matrix, which integrates information from the gene expression levels, DNA methylation and copy number aberration. Since the similarity scores from the TCGA dataset are in general skewed to 1, we first normalize the data by shifting its median around 0.5 and by bounding values that are greater than 1 and smaller than 0 to 1 and 0 respectively. We then perform *Shrinkage Clustering* to cluster the cancer samples, the result of which is shown in comparison to the true cancer types (Table 2). We can see that the algorithm generates three clusters, successfully predicting the true number of cancer types contained in the data. The clustering assignments also demonstrate high accuracy, as 98% of samples are correctly clustered with only 5 samples misclassified. In addition, we compared the performance of *Shrinkage Clustering* to that of five commonly used clustering algorithms that directly cluster similarity data: *Spectral Clustering* [31], *Hierarchical Clustering* [13] (Ward’s method [32]), *PAM* [33], *AGNES* [34], and *SymNMF* [28]. Since these five methods do not determine the optimal cluster number, the mean *Silhouette* [22] width is used to pick the optimal cluster number from a range of 2 to 10 clusters. Notably, *Shrinkage Clustering* is one of the two algorithms that estimate a three-cluster structure (with *AGNES*), and its accuracy outperforms the rest (Table 5).

**Table 2:** Clustering results of the TCGA dataset, where the clustering assignments from *Shrinkage Clustering* are compared against the three known tumor types

Tumor Type	Cluster 1	Cluster 2	Cluster 3
BRCA	3	204	0
GBM	0	0	67
LUSC	17	2	0

### Experiments on feature-based data

#### Testing with simulated and standardized data

Since similarity matrices are not always available in most clustering applications, we now test the performance of *Shrinkage Clustering* using feature-based data that does not directly provide the similarity information between objects. To run *Shrinkage Clustering*, we first convert the data to a similarity matrix using *S*= exp(−(*D*(*X*)/(*βσ*))^2^), where [*D*(*X*)]_*ij*_ is the Euclidean distance between *X*_*i*_ and *X*_*j*_, *σ* is the standard deviation of *D*(*X*), and *β*=*E*(*D*(*X*)^2^)/*σ*^2^. The same conversion method is used for all datasets in the rest of this paper.

As a proof of concept, we first generate a simulated three-cluster two-dimensional data set by sampling 50 points for each cluster from bivariate normal distributions with a common identity covariance matrix around centers at (-2, 2), (-2, 2) and (0, 2) respectively. The clustering result from *Shrinkage Clustering* is shown in Table 3, where the algorithm successfully determines the existence of 3 clusters in the data and obtains a clustering solution with high accuracy.

**Table 3:** Performances of *Shrinkage Clustering* on Simulated, Iris and Wine data, where the clustering assignments are compared against the three simulated centers, three Iris species and three wine types respectively

Simulated				Iris			Wine			
Center	C1	C2	C3	Species	C1	C2	Type	C1	C2	C3
(-2,2)	0	49	1	*setosa*	50	0	1	0	59	0
(-2,-2)	0	1	49	*versicolor*	0	50	2	59	6	0
(2,0)	50	0	0	*virginica*	0	50	3	0	6	48

Next, we test the performance of *Shrinkage Clustering* using two real data sets, the Iris [35] and the wine data [36], both of which are frequently used to test clustering algorithms; and they can be downloaded from the University of California Irvine (UCI) machine learning repository [37]. The clustering results from *Shrinkage Clustering* for both datasets are shown in Table 3, where the clustering assignments are compared to the true cluster memberships of the Iris and the wine samples respectively. In application to the wine data, *Shrinkage Clustering* successfully identifies a correct number of 3 wine types and produces highly accurate cluster memberships. For the Iris data, though the algorithm generates two instead of three clusters, the result is acceptable because the species *versicolor* and *virginica* are known to be hardly distinguishable given the features collected.

#### Case study 1: Breast Cancer Wisconsin Diagnostic (BCWD)

The BCWD dataset [38, 39] contains 569 breast cancer samples (357 benign and 212 malignant) with 30 characteristic features computed from a digitized image of a fine needle aspirate (FNA) of a breast mass. The dataset is available on the UCI machine learning repository [37] and is one of the most popularly tested dataset for clustering and classification. Here, we apply *Shrinkage Clustering* to the data and compare its performance against nine commonly used clustering methods: *Spectral Clustering* [31], *K-means* [14], *Hierarchical Clustering* [13] (Ward’s method [32]), *PAM* [33], *DBSCAN* [16], *Affinity Propagation* [40], *AGNES* [34], *clusterdp* [41], *SymNMF* [28]. Since *K-means*, *Spectral Clustering*, *Hierarchical Clustering*, *PAM*, *AGNES* and *SymNMF* do not inherently determine the optimal cluster number and require the cluster number as an input, we first run these algorithms with cluster numbers from 2 to 10, and then use the mean *Silhouette* width as the criterion to select the optimal cluster number. For algorithms that internally select the optimal cluster number (i.e. *DBSCAN*, *Affinity Propagation* and *clusterdp*), we tune the parameters to generate clustering solutions with cluster numbers similar to the true cluster numbers so that the accuracy comparison is less biased. The parameter values for each algorithm are specified in Table 4. For *DBSCAN*, the clustering memberships of non-noise samples are used for assessing accuracy. The accuracy of all clustering solutions is evaluated using four metrics: Normalized Mutual Information (NMI) [42], Rand Index [42], F1 score [42], and the optimal cluster number (K).

**Table 4:** Parameter values of *DBSCAN*, *Affinity Propagation* and *clusterdp*

Algorithm	DBSCAN		Affinity propagation		clusterdp	
Parameter	minPts	eps	p	q	rho	delta
BCWD	31	3000	NA	0	20	3000
Dyrskjot-2003	2	23000	NA	0.07	3	20000
Nutt-2003-v1	2	11000	NA	0.12	1.5	3000
Nutt-2003-v3	1	8000	NA	0.1	1	7000
AIBT	5	400	NA	0	2.5	240

**Table 5:** Performance comparison of ten algorithms on six biological data sets, i.e. TCGA, BCWD, Dyrskjot-2003, Nutt-2003-v1, Nutt-2003-v3 and AIBT

Data	Metric	Shrinkage	Spectral	K-means	Hierarchical	PAM	DBSCAN	Affinity	AGNES	Clusterdp	SymNMF
TCGA	NMI	**0.91**	0.77	NA	0.83	0.76	NA	NA	**0.82**	NA	**0.78**
	Rand	**0.97**	0.91	NA	0.91	0.77	NA	NA	**0.90**	NA	**0.94**
	F1	**0.98**	0.92	NA	0.92	0.80	NA	NA	**0.92**	NA	**0.95**
	K (3)	**3**	2	NA	2	2	NA	NA	**3**	NA	2
BCWD	NMI	**0.50**	0.29	0.46	0.09	**0.50**	0.20	0.45	0.09	0.20	**0.56**
	Rand	**0.77**	0.68	0.75	0.55	**0.77**	0.64	0.76	0.55	0.53	**0.83**
	F1	**0.80**	0.69	0.79	0.69	**0.80**	0.75	0.79	0.69	0.59	**0.85**
	K (2)	**2**	2	2	2	**2**	2	3	2	2	**2**
Dyrskjot-2003	NMI	**0.45**	0.07	**0.51**	0.12	0.56	0.30	0.42	0.12	0.07	**0.58**
	Rand	**0.78**	0.55	**0.76**	0.42	0.77	0.55	0.72	0.42	0.50	**0.83**
	F1	**0.70**	0.36	**0.71**	0.54	0.66	0.60	0.66	0.54	0.43	**0.75**
	K (3)	**3**	3	**3**	3	3	3	3	3	2	**3**
Nutt-2003-v1	NMI	**0.56**	0.45	0.47	0.28	0.34	**0.61**	0.41	0.11	0.17	**0.49**
	Rand	**0.72**	0.73	0.72	0.52	0.68	**0.65**	0.73	0.35	0.64	**0.72**
	F1	**0.58**	0.51	0.51	0.43	0.41	**0.62**	0.44	0.38	0.34	**0.55**
	K (4)	**4**	4	4	4	4	**4**	5	4	4	**4**
Nutt-2003-v3	NMI	**1.00**	0.20	**0.75**	0.13	0.33	0.13	0.13	0.13	0.29	**0.76**
	Rand	**1.00**	0.58	**0.91**	0.58	0.58	0.58	0.58	0.58	0.55	**0.91**
	F1	**1.00**	0.59	**0.92**	0.71	0.60	0.71	0.71	0.71	0.57	**0.91**
	K (2)	**2**	2	**2**	2	2	2	3	2	2	**2**
AIBT	NMI	**0.56**	0.20	**0.58**	0.17	0.54	0.56	0.53	0.02	0.55	**0.55**
	Rand	**0.79**	0.68	**0.80**	0.37	0.78	0.65	0.76	0.26	0.69	**0.79**
	F1	**0.61**	0.39	**0.62**	0.40	0.59	0.59	0.51	0.40	0.57	**0.61**
	K (4)	**4**	4	**4**	4	4	4	5	4	3	**4**

Clustering accuracy is assessed via metrics including NMI (Normalized Mutual Information), Rand Index, F1 score and K (the optimal cluster number). The top three performers in each case are highlighted in bold

The performance results (Table 5) show that *Shrinkage Clustering* correctly predicts a 2 cluster structure from the data and generates the clustering assignments with high accuracy. When comparing the cluster assignments against the true cluster memberships, we can see that *Shrinkage Clustering* is among the top three best performers across all accuracy metrics.

#### Case study 2: Benchmarking gene expression data for cancer subtyping

Next, we test the performance of *Shrinkage Clustering* as well as the nine commonly used algorithms in application to identifying cancer subtypes using three benchmarking datasets from de Souto et al. [43]: Dyrskjot-2003 [44], Nutt-2003-v1 [45] and Nutt-2003-v3 [45]. Dyrskjot-2003 contains the expression levels of 1203 genes in 40 well-characterized bladder tumor biopsy samples from three subclasses of bladder carcinoma: T2+ (9 samples), Ta (20 samples), and T1 (11 samples). Nutt-2003-v1 contains the expression levels of 1377 genes in 50 gliomas from four subclasses: classic gliobalstomas (14 samples), classic anaplastic oligodendrogliomas (7 samples), nonclassic glioblastomas (14 samples), and nonclassic anaplastic oligodendrogliomas (15 samples). Nutt-2003-v3 is a subset of Nutt-2003-v1, containing 7 samples of classic anaplastic oligodendrogliomas and 15 samples of nonclassic anaplastic oligodendrogliomas with the expression of 1152 genes. All three data sets are small in sample sizes and high in dimensions, which is often the case in clinical research. The performance of all ten algorithms is compared using the same metrics as in the previous case study, and the result is shown in Table 5. Though there is no clear winning algorithm across all data sets, *Shrinkage Clustering* is among the top three performers in all cases, along with other top performing algorithms such as *SymNMF*, *K-means* and *DBSCAN*. Since the clustering results from *DBSCAN* are compared to the true cluster assignments excluding the noise samples, the accuracy of *DBSCAN* may be slightly overestimated.

#### Case Study 3: Allen Institute Brain Tissue (AIBT)

The AIBT dataset [46] contains RNA sequencing data of 377 samples from four types of brain tissues, i.e. 99 samples of temporal cortex, 91 samples of parietal cortex, 93 samples of cortical white matter, and 94 samples hippocampus isolated by macro-dissection. For each sample, the expression levels of 50282 genes are included as features, and each feature is normalized to have a mean of 0 and a standard deviation of 1 prior to testing. In contrast to the previous case study, the AIBT data is much larger in size with significantly more features being measured. Therefore, this would be a great example to test both the accuracy and the speed of clustering algorithms in face of greater data sizes and higher dimensions.

Similar to the previous case studies, we apply *Shrinkage Clustering* and the nine commonly used clustering algorithms to the data, and use mean *Silhouette* width to select the optimal cluster number for algorithms that do not inherently determine the cluster number. The performances of all ten algorithms measured across the four accuracy metrics (i.e. NMI, Rand, F1, K) are shown in Table 5. We can see that *Shrinkage Clustering* is the second best performer among all ten algorithms in terms of clustering quality, with comparable accuracy to the top performer (*K-means*).

**Fig. 4:**
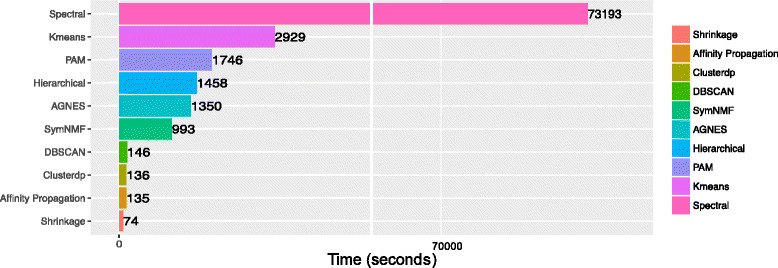
Speed comparison using the AIBT data. The computation time of *Shrinkage Clustering* is recorded and compared against other commonly used clustering algorithms

Next, we record and compare the speed of the ten algorithms for clustering the data. The speed comparison results, shown in Fig. 4, demonstrate the unparalleled speed of *Shrinkage Clustering* compared to the rest of the algorithms. Compared to algorithms that automatically select optimal number of clsuters (*DBSCAN*, *Affinity Propagation* and *Clusterdp*), *Shrinkage Clustering* is two times faster in speed; compared to algorithms that are coupled with external cluster validation algorithms for cluster number selection, *Shrinkage Clustering* is at least 14 times faster. In particular, the same data that takes *Shrinkage Clustering* only 73 s to cluster can take *Spectral clustering* more than 20 h.

## Discussion

From the biological case studies, we showed that *Shrinkage Clustering* is computationally advantageous in speed with comparable clustering accuracy to top performing clustering algorithms and higher clustering accuracy than algorithms that internally select cluster numbers. The advantage in speed mainly comes from the fact that *Shrinkage Clustering* integrates the clustering of the data and the determination of the optimal cluster number into one seamless process, so the algorithm only needs to run once in order to complete the clustering task. In contrast, algorithms like *K-means*, *PAM*, *Spectral Clustering*, *AGNES* and *SymNMF* perform clustering on a single cluster number basis, therefore they need to be repeatedly run for all cluster numbers of interest before a clustering evaluation method can be applied. Notably, the clustering evaluation method *Silhouette* that we used in this experiment does not perform any repetitive clustering validation and therefore is a much faster method compared to other commonly used methods that require repetitive validation [27]. This means that *Shrinkage Clustering* would have an even greater advantage in computation speed compared to the methods tested in this paper if we use a cluster evaluation method that has a repetitive nature (e.g. *Consensus Clustering*, *Gap Statistics*, *Stability Selection*).

One prominent feature of *Shrinkage Clustering* is its flexibility to add the constraint of minimum cluster sizes. The size constraints can help prevent generating empty or tiny clusters (which are often observed in *Hierarchical Clustering* and sometimes in *K-means* applications), and can produce clusters of sufficiently large sample sizes as required by the user. This is particularly useful when we need to perform subsequent statistical analyses based on the clustering solution, since clusters of too small a size can make a statistical testing infeasible. For example, one application of cluster analysis in clinical studies is identifying subpopulations of cancer patients based on their gene expression levels, which is usually followed with a survival analysis to determine the prognostic value of the gene expression patterns. In this case, clusters that contain too few patients can hardly generate any significant or meaningful patient outcome comparison. In addition, it is difficult to take actions based on tiny patient clusters (e.g. in the context of designing clinical trials), because these clusters are hard to validate. Since adding minimum size constraints is essentially merging tiny clusters into larger ones and might result in less homogeneous clusters, this approach is unfavorable if the researcher wishes to identify the outliers in the data or to obtain more homogeneous clusters. In these scenarios, we would recommend using the base algorithm without adding the minimum size constraint.

Despite its superior speed and high accuracy, *Shrinkage Clustering* has a couple of limitations. First, the automatic convergence to an optimal cluster number is a double-edged sword. This feature helps to determine the optimal cluster number and speeds up the clustering process dramatically, however it can be unfavorable when the researcher has a desired cluster number in mind that is different from the cluster number identified by the algorithm. Second, the algorithm is based on the assumption of hard clustering, therefore it currently does not provide probabilistic frameworks as those offered by soft clustering. In addition, due to the similarity between *symNMF* and *K-means*, the algorithm likely prefers spherical clusters if the similarity matrix is derived from Euclidean distances. Interesting future research directions include exploring and extending the capability of *Shrinkage Clustering* to identify oddly-shaped clusters, to deal with missing data or incomplete similarity matrices, as well as to handle semi-supervised clustering tasks with must-link and cannot-link constraints.

## Conclusions

In summary, we developed a new NMF-based clustering method, *Shrinkage Clustering*, which shrinks the number of clusters to an optimum while simultaneously optimizing the cluster memberships. The algorithm performed with high accuracy on both simulated and actual data, exhibited excellent robustness to noise, and demonstrated superior speeds compared to some of the commonly used algorithms. The base algorithm has also been extended to accommodate requirements on minimum cluster sizes, which can be particularly beneficial to clinical studies and the general biomedical community.

## References

[1] Sørlie T, Tibshirani R, Parker J, Hastie T, Marron J, Nobel A (2003). Repeated observation of breast tumor subtypes in independent gene expression data sets. Proc Natl Acad Sci.

[2] Wirapati P, Sotiriou C, Kunkel S, Farmer P, Pradervand S, Haibe-Kains B (2008). Meta-analysis of gene expression profiles in breast cancer: toward a unified understanding of breast cancer subtyping and prognosis signatures. Breast Cancer Res.

[3] Rouzier R, Perou CM, Symmans WF, Ibrahim N, Cristofanilli M, Anderson K (2005). Breast cancer molecular subtypes respond differently to preoperative chemotherapy. Clin Cancer Res.

[4] Abascal F, Valencia A (2002). Clustering of proximal sequence space for the identification of protein families. Bioinformatics.

[5] Stam MR, Danchin EG, Rancurel C, Coutinho PM, Henrissat B (2006). Dividing the large glycoside hydrolase family 13 into subfamilies: towards improved functional annotations of *α*-amylase-related proteins. Protein Eng Des Sel.

[6] de Lima EB, Júnior WM, de Melo-Minardi RC (2016). Isofunctional Protein Subfamily Detection Using Data Integration and Spectral Clustering. PLoS Comput Biol.

[7] Chen X, Velliste M, Weinstein S, Jarvik JW, Murphy RF. Location proteomics—Building subcellular location tree from high resolution 3D fluorescence microcope images of randomly-tagged proteins. Manipulation and Analysis of Biomolecules, Cells, and Tissues, Proceedings of SPIE 4962; 2003, pp. 298–306.

[8] Slater JH, Culver JC, Long BL, Hu CW, Hu J, Birk TF (2015). Recapitulation and modulation of the cellular architecture of a user-chosen cell of interest using cell-derived, biomimetic patterning. ACS nano.

[9] Haldar P, Pavord ID, Shaw DE, Berry MA, Thomas M, Brightling CE (2008). Cluster analysis and clinical asthma phenotypes. Am J Respir Crit Care Med.

[10] Moore WC, Meyers DA, Wenzel SE, Teague WG, Li H, Li X (2010). Identification of asthma phenotypes using cluster analysis in the Severe Asthma Research Program. Am J Respir Crit Care Med.

[11] Jain AK, Murty MN, Flynn PJ (1999). Data clustering: a review. ACM Comput Surv (CSUR).

[12] Wiwie C, Baumbach J, Röttger R (2015). Comparing the performance of biomedical clustering methods. Nat Med.

[13] Johnson SC (1967). Hierarchical clustering schemes. Psychometrika.

[14] MacQueen J (1967). Some methods for classification and analysis of multivariate observations. Proceedings of the fifth Berkeley symposium on mathematical statistics and probability, vol. 1, No. 14.

[15] Lloyd S (1982). Least squares quantization in PCM. Inf Theory IEEE Trans.

[16] Ester M, Kriegel HP, Sander J, Xu X. A density-based algorithm for discovering clusters in large spatial databases with noise. In: KDD. vol. 96, No. 34. Portland: 1996. p. 226–31.

[17] McLachlan GJ, Basford KE (1988). Mixture models: inference and applications to clustering.

[18] Shi J, Malik J (2000). Normalized cuts and image segmentation. Pattern Anal Mach Intell IEEE Trans.

[19] Li T, Ding CH (2013). Data Clustering: Algorithms and Applications.

[20] Ding C, He X, Simon HD (2005). On the equivalence of nonnegative matrix factorization and spectral clustering. Proceedings of the 2005 SIAM International Conference on Data Mining.

[21] Brunet JP, Tamayo P, Golub TR, Mesirov JP (2004). Metagenes and molecular pattern discovery using matrix factorization. Proc Natl Acad Sci.

[22] Rousseeuw PJ (1987). Silhouettes: a graphical aid to the interpretation and validation of cluster analysis. J Comput Appl Math.

[23] Pelleg D, Moore AW (2000). X-means: Extending K-means with Efficient Estimation of the Number of Clusters. ICML ’00 Proceedings of the Seventeenth International Conference on Machine Learning.

[24] Tibshirani R, Walther G, Hastie T (2001). Estimating the number of clusters in a data set via the gap statistic. J R Stat Soc Ser B Stat Methodol.

[25] Monti S, Tamayo P, Mesirov J, Golub T (2003). Consensus clustering: a resampling-based method for class discovery and visualization of gene expression microarray data. Mach Learn.

[26] Lange T, Roth V, Braun ML, Buhmann JM (2004). Stability-based validation of clustering solutions. Neural Comput.

[27] Hu CW, Kornblau SM, Slater JH, Qutub AA. Progeny Clustering: A Method to Identify Biological Phenotypes. Sci Rep. 2015; 5(12894):5. 10.1038/srep12894. 10.1038/srep12894 PMC4533525 26267476

[28] Kuang D, Ding C, Park H (2012). Symmetric nonnegative matrix factorization for graph clustering. Proceedings of the 2012 SIAM international conference on data mining.

[29] Bradley P, Bennett K, Demiriz A (2000). Constrained k-means clustering.

[30] Speicher N, Lengauer T (2012). Towards the identification of cancer subtypes by integrative clustering of molecular data.

[31] Zeileis A, Hornik K, Smola A, Karatzoglou A (2004). kernlab-an S4 package for kernel methods in R. J Stat Softw.

[32] Ward Jr JH (1963). Hierarchical grouping to optimize an objective function. J Am Stat Assoc.

[33] Maechler M, Rousseeuw P, Struyf A, Hubert M, Hornik K (2012). Cluster: cluster analysis basics and extensions. R Package Version.

[34] Kaufman L, Rousseeuw PJ (2009). Finding groups in data: an introduction to cluster analysis, vol. 344..

[35] Fisher RA (1936). The use of multiple measurements in taxonomic problems. Ann Eugenics.

[36] Aeberhard S, Coomans D, De Vel O. Comparison of classifiers in high dimensional settings. Dept Math Statist, James Cook Univ, North Queensland, Australia. Tech Rep. 1992;92-02.

[37] Bache K, Lichman M. UCI Machine Learning Repository: University of California, Irvine, School of Information and Computer Sciences; 2013. http://archive.ics.uci.edu/ml.

[38] Street WN, Wolberg WH, Mangasarian OL (1993). Nuclear feature extraction for breast tumor diagnosis. IS&T/SPIE’s Symposium on Electronic Imaging: Science and Technology.

[39] Mangasarian OL, Street WN, Wolberg WH (1995). Breast cancer diagnosis and prognosis via linear programming. Oper Res.

[40] Frey BJ, Dueck D (2007). Clustering by passing messages between data points. Science.

[41] Rodriguez A, Laio A (2014). Clustering by fast search and find of density peaks. Science.

[42] Manning CD, Raghavan P, Schütze H (2008). Introduction to information retrieval, vol. 1..

[43] de Souto MC, Costa IG, de Araujo DS, Ludermir TB, Schliep A (2008). Clustering cancer gene expression data: a comparative study. BMC Bioinformatics.

[44] Dyrskjøt L, Thykjaer T, Kruhøffer M, Jensen JL, Marcussen N, Hamilton-Dutoit S (2003). Identifying distinct classes of bladder carcinoma using microarrays. Nat Genet.

[45] Nutt CL, Mani D, Betensky RA, Tamayo P, Cairncross JG, Ladd C (2003). Gene expression-based classification of malignant gliomas correlates better with survival than histological classification. Cancer Res.

[46] Montine JT, Sonnen AJ, Montine SK, Crane KP, Larson BE (2012). Adult Changes in Thought study: dementia is an individually varying convergent syndrome with prevalent clinically silent diseases that may be modified by some commonly used therapeutics. Curr Alzheim Res.

